# Sex-specific prevalence, awareness, treatment and control of hypertension in adults in India: a study for developing sex-specific public policy from the longitudinal ageing study in India (LASI) data 2017–2018

**DOI:** 10.1186/s41043-023-00404-3

**Published:** 2023-08-25

**Authors:** Ayushi Singh, Priyanka Dixit

**Affiliations:** 1 Present Address: School of Health Systems Studies (SHSS), Tata Institute of Social Sciences (TISS), V. N. Purav Marg, Deonar, Mumbai 400088 India; 2 Present Address: Centre for Health and Social Sciences, School of Health Systems Studies (SHSS), Tata Institute of Social Sciences (TISS), V. N. Purav Marg, Deonar, Mumbai 400088 India

**Keywords:** Sex, Hypertension, India, Longitudinal Ageing Study in India-2017–2018

## Abstract

**Background and objective:**

Hypertension is a key risk factor for cardiovascular disease and the leading cause of mortality among Indian adults. The difference in health status between men and women is becoming a great burden in itself worldwide. This study aimed to examine the differences between men and women in the prevalence, awareness, treatment, and control of hypertension and related risk factors among people aged 45 and older in India using data from the Longitudinal Ageing Study in India in 2017–2018.

**Methods:**

Descriptive statistics were presented separately for males and females. Multivariable logistic regression was used to analyze the socio-demographic, lifestyle behaviours, and biological factors associated with the prevalence of hypertension. All statistical analyses were conducted using Stata Version 16.0 statistical software. The study of the data was conducted using survey weights available in the LASI datasets.

**Key findings:**

Overall, the study found that 45.1% of the study population had hypertension, with 26.9% self-reporting their condition and 30% having hypertension at the time of measurement. Approximately 41% of males and 59% of females had hypertension. The self-reported hypertension of men was found to differ significantly from measured hypertension by 8.7%, while in women the difference was only 1.2%. Diabetes was found to increase the odds of having hypertension in both males (OR = 3.65, 95% CI (3.37–3.97)) and females (OR = 3.46, 95% CI (3.21–3.74)).

**Conclusion:**

The difference between self-reported and measured hypertension in men and women is contributing to sex-gender and health inequalities that must be addressed. For adult females with hypertension, it is important to prioritize obesity, education level, physical activity, and regular clinic visits to manage chronic conditions. Based on our findings, policy recommendations can be made to focus on increasing women's literacy, promoting men's screening for hypertension, banning tobacco and alcohol sales, and organizing hypertension awareness campaigns specifically for men and in rural areas.

## Background

India is seeing an upsurge in Non-Communicable Diseases (NCDs) while also dealing with a high burden of infectious diseases and maternal and child health issues [1]. Elevated blood pressure, or hypertension, is the most significant contributor to the global burden of disease and mortality, leading to approximately 9.4 million deaths annually [2]. Also, cardiovascular disease (CVD) is the leading cause of NCD mortality, accounting for nearly 44% of all fatalities [3]. The Global Burden of Hypertension (GBD) study has highlighted the global burden of 212 million disability-adjusted life years (DALYs) related to hypertension, of which 18% occurred in India in 2015 [4]. Approximately 972 million people worldwide, or 26% of the total population, have hypertension. This prevalence is anticipated to increase to 29% by 2025, mostly due to the high rate of increase in economically developing countries [5]. The prevalence of hypertension is expected to rise to 44% (with a confidence interval of 43–45%) by 2030, which represents a 17% increase instead of the 25% decrease projected by the World Health Organisation for the same year[6].

In 2019, approximately 32% of women and 34% of men between the ages of 30 and 79 worldwide were diagnosed with hypertension [7]. Traditionally, men have a more significant incidence of total cardiovascular morbidities at all ages; hypertension in women has received little attention in the past compared to their male counterparts [8]. In India, according to the National Family Health Survey (NFHS-5) conducted in 2019–2021, the occurrence of hypertension in men was found to be 24%, and in women, it was 21%. This is an increase from the previous round conducted in 2015–2016, where the prevalence was 19% and 17% in men and women, respectively [9]. Roy and his colleagues (2017), found that the prevalence of hypertension in India has risen dramatically (23–42.2% in urban areas and 11.2–28.9% in rural areas) over the last two decades. They added that awareness, treatment, and control of hypertension had not significantly improved [10].

India is currently undergoing an epidemiological transition in which it is experiencing unprecedented demographic changes. Increased longevity and falling fertility have led to a dramatic increase in the population of elderly people aged 60 and older. Growing older was linked to hypertension, as in Ethiopia, the prevalence of hypertension increased from 9.5% in the 18–25 age group to 46.3% in those above 65 years [11]. In a study on people aged 80 years and older in urban south India, the prevalence of hypertension was 83.5% [12]. While hypertension control has improved globally in recent decades, the prevalence of hypertension has surged in low- and middle-income countries (LMICs), with low levels of hypertension awareness, treatment, and control [13].

In India, a significant proportion of hypertension patients are unaware of their condition. A majority of hypertensive patients in rural India and a significant proportion of those in urban India are unaware of their hypertension. Among those diagnosed with hypertension, only a quarter in rural India and a third in urban India receive therapy [14]. Less than half (42%) of individuals with hypertension are diagnosed and treated, with an estimated 46% of adults with hypertension being unaware of their disease [3]. According to the WHO, 1 in 4 men and 1 in 5 women had hypertension in 2015, and less than 1 in 5 people with hypertension have the problem under control [15]. NCDs are known to develop during middle age as a result of prolonged exposure to unhealthy lifestyle habits, including tobacco use, alcohol consumption, a lack of regular physical activity, and the consumption of diets high in saturated fats, sugars, and salt, such as fast foods. This type of lifestyle leads to an increase in risk factors such as hypertension, dyslipidemia, diabetes, and obesity, which can act both independently and in combination with each other [16].

Previous studies on hypertension in India have focused primarily on small-scale community or hospital settings and have been limited in scope, with very few studies conducted from 1999 to 2020 exploring the prevalence, awareness, treatment, and control of hypertension in specific states of India. Furthermore, there is a dearth of research on hypertension, sex differences in hypertension, and its associated determinants among older adults in India. To date, there has been a national-level study in India that provides information on sex differences in the treatment, awareness, and control of hypertension. As a result, there is a lack of relevant data available to develop sex-specific public policies in the context of hypertension. This research aims to address this gap by investigating the prevalence, awareness, treatment, control, and related determinants of hypertension based on sex in adults in India, with the objective of designing a specific public policy to reduce the burden of hypertension in India.

## Materials and methods

### Study setting and participants

The data for this cross-sectional research came from the first wave of the LASI. The LASI is a large-scale countrywide study of India's health, economic, and social variables, as well as the consequences of population ageing. The LASI is a biennial panel survey of India's (states & Union territories) adult and older population. The LASI survey's major goal was to produce scientific data on demographics, household economic position, functional and mental health, chronic health issues, biomarkers, health care usage, work, employment, and so on. "To arrive at the final units of observation, the LASI used a multistage stratified area probability cluster sampling design" [17]. The overall sample size for this study was 72,250, which included all eligible older individuals aged 18 and above.

### Measures

#### Outcome variables

In LASI, there is a section on biomarker measurements, in which the blood pressure of older adults is measured. "It was taken in a sitting position using a digital sphygmomanometer (Omron® HEM 7121 BP monitor) on the left mid-arm at heart level, after resting for five minutes. Three blood pressure measurements were taken at five-minute intervals, and the mean value of the last two measurements was calculated. An individual was considered to be hypertensive if their systolic blood pressure (SBP) was ≥ 140 mmHg or diastolic blood pressure (DBP) ≥ 90 mmHg" [17]. We used the concept of ALL hypertension, which is calculated as follows: First, we identified individuals with self-reported hypertension, as in LASI. Self-reported hypertension was assessed by asking the question, "Has any health professional ever told you that you have hypertension or high blood pressure?" Only those who answered "Yes" to the relevant question were classified as hypertensive. We took the respondents who said "No" to the above question and measured hypertensive (i.e., systolic > 140mmhg and diastolic > 90mmhg). After that, we combined those who answered YES to the self-reported hypertension question and those who were measured hypertensive (from No population) to find ALL hypertensive respondents. Only those who identified themselves as self-reported hypertensive were asked about their treatment-seeking behaviour. "In order to control your blood pressure or hypertension, are you currently taking any medication?" People with measured hypertension who had never received information about it from a doctor or other healthcare provider were regarded as being unaware of their condition. Those individuals who self-reported their hypertensive condition and measured hypertensive were considered aware of their condition. We determined the prevalence of the control of hypertension as the percentage of people who stated that they had been diagnosed with the condition by a medical practitioner and whose measured systolic and diastolic blood pressures were less than 140 and 90 mmHg, respectively.

### Covariates

#### Socio-demographic variables

The LASI collected information regarding various socio-demographic variables such as age: "18–44, 45–54, 55–64, 65–74, or 75 + years" and sex: "male or female." Educational level: "no education, primary, secondary, or higher", working status: "never worked, currently working, or not currently working," and marital status: "currently married, widowed, divorced, separated, or deserted." LASI collected data on household spending on food ("a 7-day reference period") and non-food products ("reference periods of 30 and 365 days") at the household level. After "standardising food and non-food spending to a 30-day reference period, the monthly per capita consumption expenditure (MPCE) was computed". The MPCE was used as a summary indicator of consumption as "poorest, poorer, middle, richer, and richest." Also, other necessary components of household factors were incorporated: a place of residence: "rural or urban," region: "North, Central, East, Northeast, West, and South," religion: "Hindu, Muslim, Christian, or other," caste: "scheduled tribe, scheduled caste, other backward class, and other." These variables were all taken into account in this study.

### Lifestyle behaviours

Respondents in the LASI were asked about their tobacco usage status: "smoking or smokeless." Based on their responses to this question, the participants were divided into three groups: "never smokers, former smokers, and current smokers." A yes-or-no question was used to determine whether or not the person drank alcohol.

The physical activity indicators for the LASI were created based on the WHO's worldwide guidelines for moderate and vigorous physical activity [18]. They further divided the physical activity into two categories: Moderate- “cleaning the house, washing clothes by hand, fetching water or wood, drawing water from a well, gardening, bicycling at a regular pace, walking at a moderate pace, dancing, floor or stretching exercises” and Vigorous- “running or jogging, swimming, going to a health centre or gym, cycling, or digging with a spade or shovel, heavy lifting, chopping, farm work, fast bicycling, cycling with loads”. The possible responses for both the activities were- “every day, more than once a week, once a week, one to three times per month, and hardly ever or never.” Based on these responses, we classified the respondent as “physically active” (“more than once a week”) and “physically inactive” (“once a week or less often”) for moderate and vigorous activities.

### Health status

In this study, from LASI, we have included three self-reported chronic diseases that are diagnosed by the doctor or any health professional: Diabetes, Arthritis, and Stroke. Body mass index (BMI) was recorded as "Underweight"—(< 18.5), "Normal"—(18.5–24.9), "Overweight"—(25–29.9), or "Obese"—(30 and above). For the sake of analysis, we have merged the terms overweight and obese. The terms "functioning" and "disability" refer to various aspects of a person's physical and mental abilities. This includes the functioning of the body and its structures, any limitations that people may experience when performing activities, and their level of involvement in all aspects of life. Additionally, the concept also takes into account how environmental factors impact these experiences and whether they serve as facilitators or barriers [19]. Basic and instrumental activities of daily living were used to measure functional health. Six basic ADLs (BADLs) include "dressing, indoor mobility, bathing, eating difficulties, getting in or out of bed, and using the toilet," and seven instrumental ADLs (IADLs): "food preparation, shopping for groceries, taking medication, making telephone calls, doing work around the house or garden, ability to handle finances, and getting around or finding an address in unfamiliar places." For measuring the functional limits, we set up two variables: "difficulty in ADLs" (at least one difficulty in six BADLs) and "difficulty in IADLs" (at least one difficulty in seven IADLs) [20].

### Statistical analysis

We presented the descriptive background characteristics table, i.e., the profile table of socio-demographics, lifestyle behaviour, and health status by “overall sample” people who were not having hypertension and ALL hypertension (Table 1). Further adjusted odds ratios (AORs) with 95 percent confidence intervals (CIs) were calculated for factors affecting hypertension prevalence, awareness, treatment, and control using multiple logistic regression models, separately for males and females (Tables 2 and 3). To determine how Prevalence, Awareness, Treatment, and Control of hypertension are associated with different factors such as age, education, working status, marital status, morbidities (diabetes, stroke, and arthritis), lifestyle factors (smoking status, chewing tobacco, alcohol consumption, moderate and vigorous activities), and household factors (MPCE quintile, religion, caste, and residence), we conducted a multivariable logistic regression analysis. Due to the distinct burden of hypertension and the distribution of its determinants in both sexes, all analyses were conducted separately for men and women.

**Table 1:** Characteristics of study participants by hypertension levels of older adults by sex, India, LASI Wave 1, 2017–18

Individual factors	Overall (n = 72,250)	No hypertension (n = 53,497)	No hypertension		All hypertension (Overall)	All hypertension	
			Males	Females	(n = 32,635)	Males	Females
			(n = 23,246)	(n = 30,251)		(n = 13,387)	(n = 19,247)
Age groups							
18–44	6,276 (8.69%)	5,358 (10.02%)	28 (0.12%)	5,291 (17.62%)	1,718 (5.27%)	13 (0.10%)	1,669 (8.86%)
45–54	23,058 (31.91%)	18,411 (34.42%)	8,640 (36.8%)	9,785 (32.85%)	8,833 (27.07%)	4,045 (29.31%)	4,803 (25.51%)
55–64	19,684 (27.25%)	14,373 (26.87%)	6,927 (29.51%)	7,459 (24.84%)	9,278 (28.43%)	4,021 (29.13%)	5,262 (27.94%)
65–74	15,693 (21.72%)	10,466 (19.57%)	5,352 (22.8%)	5,129 (17.08%)	8,680 (26.6%)	3,908 (28.32%)	4,784 (25.40%)
75 +	7,536 (10.43%)	4,887 (9.14%)	2,529 (10.77%)	2,365 (7.88%)	4,123 (12.63%)	1,814 (13.14%)	2,312 (12.28%)
Test of Significance			p=0.00	p=0.00		p=0.00	p=0.00
Education level							
No education	35,761 (49.5%)	27,578 (51.55%)	8,531 (36.34%)	18,993 (63.25%)	15,282 (46.83%)	4,095 (29.67%)	11,065 (58.76%)
Primary	16,769 (23.21%)	12,200 (22.81%)	6,676 (28.44%)	5,550 (18.48%)	7,827 (23.99%)	3,958 (28.68%)	3,902 (20.72%)
Secondary	12,215 (16.91%)	8,485 (15.86%)	5,098 (21.72%)	3,412 (11.36%)	5,719 (17.52%)	3,283 (23.79%)	2,479 (13.17%)
Higher	7,498 (10.38%)	5,230 (9.78%)	3,172 (13.51%)	2,074 (6.91%)	3,805 (11.66%)	2,464 (17.86%)	1,384 (7.35%)
Test of Significance			p=0.00	p=0.00		p=0.00	p=0.00
Currently working							
Never worked	19,915 (27.57%)	13,511 (25.26%)	684 (2.92%)	12,741 (42.43%)	9,779 (29.97%)	418 (3.03%)	9,170 (48.70%)
Currently working	33,430 (46.28%)	27,480 (51.37%)	16,497 (70.27%)	11,065 (36.85%)	12,830 (39.32%)	8,004 (57.99%)	4,958 (26.33%)
Not currently working	18,883 (26.14%)	12,500 (23.37%)	6,294 (26.81%)	6,222 (20.72%)	10,022 (30.71%)	5,379 (38.98%)	4,701 (24.97%)
Test of Significance			p=0.00	p=0.00		p=0.00	p=0.00
Marital status							
Currently married	54,620 (75.6%)	41,538 (77.65%)	20,430 (87.02%)	21,156 (70.45%)	23,148 (70.93%)	11,921 (86.38%)	11,335 (60.19%)
Widowed	15,650 (21.66%)	10,375 (19.39%)	2,346 (9.99%)	7,994 (26.62%)	8,705 (26.68%)	1,526 (11.06%)	7,069 (37.54%)
D/S/D/Others^a^	1,974 (2.73%)	1,582 (2.96%)	701 (2.99%)	881 (2.94%)	779 (2.39%)	353 (2.56%)	427 (2.27%)
Test of Significance			p=0.00	p=0.00		p=0.00	p=0.00
Morbidities							
Diabetes							
No	63,724 (88.44%)	50,409 (94.23%)	21,916 (93.35%)	28,503 (94.91%)	26,241 (80.42%)	10,913 (79.07%)	15,318 (81.35%)
Yes	8,330 (11.56%)	3,085 (5.77%)	1,561 (6.65%)	1,528 (5.09%)	6,391 (19.58%)	2,888 (20.93%)	3,512 (18.65%)
Test of Significance			p=0.00	p=0.00		p=0.00	p=0.00
Stroke							
No	70,759 (98.19%)	52,965 (99.01%)	23,419 (98.6%)	29,828 (99.33%)	31,686 (97.10%)	13,261 (96.08%)	18,417 (97.81%)
Yes	1,304 (1.81%)	530 (0.99%)	329 (1.40%)	202 (0.67%)	945 (2.60%)	540 (3.92%)	412 (2.19%)
Test of Significance			p=0.00	p=0.00		p=0.00	p=0.00
Arthritis							
No	65,646 (91.09%)	49,585 (92.69%)	21,987 (93.26%)	27,703 (92.25%)	29,131 (89.27%)	12,752 (92.40%)	16,400 (87.09%)
Yes	6,419 (8.91%)	3,911 (7.31%)	1,581 (6.74%)	2,328 (7.75%)	3,502 (10.73%)	1,049 (7.60%)	2,431 (12.91%)
Test of Significance			p=0.00	p=0.00		p=0.00	p=0.00
Difficulty in ADL^b^							
No	60,490 (84.08%)	45,941 (86.04%)	20,454 (87.28%)	25,503 (85.08%)	26,319 (80.73%)	11,360 (82.4%)	14,970 (79.58%)
Yes	11,452 (15.92%)	7,456 (13.96%)	2,980 (12.72%)	4,473 (14.92%)	6,280 (19.27%)	2,426 (17.6%)	3,842 (20.42%)
Test of Significance			p=0.00	p=0.00		p=0.00	p=0.00
Difficulty in IADL^c^							
No	45,910 (63.87%)	35,276 (66.11%)	17,223 (73.54%)	18,091 (60.40%)	19,636 (60.28%)	9,529 (69.15%)	10,170 (54.11%)
Yes	25,972 (36.13%)	18,080 (33.89%)	6,197 (26.46%)	11,859 (39.6%)	12,938 (39.72%)	4,250 (30.85%)	8,624 (45.89%)
Test of Significance			p=0.00	p=0.00		p=0.00	p=0.00
Lifestyle factors							
BMI categories							
Normal	33,453 (51.32%)	25,941 (53.27%)	12,178 (57.02%)	13,777 (50.37%)	14,707 (48.29%)	7,059 (54.84%)	7,693 (43.76%)
Underweight	13,454 (20.64%)	11,510 (23.64%)	5,442 (25.48%)	6,075 (22.21%)	4,371 (14.35%)	1,873 (14.56%)	2,498 (14.21%)
Overweight/obese	18,274 (28.04%)	11,248 (23.10%)	3,739 (17.51%)	7,497 (27.41%)	11,375 (37.35%)	3,939 (30.60%)	7,389 (42.03%)
Test of Significance			p=0.00	p=0.00		p=0.00	p=0.00
Moderate activities							
Inactive	25,850 (35.71%)	18,194 (34.22%)	10,550 (45.25%)	7,692 (25.76%)	12,510 (38.49%)	6,788 (49.41%)	5,799 (30.91%)
Active	46,044 (64.29%)	34,970 (65.78%)	12,767 (54.75%)	22,169 (74.24%)	19,991 (61.51%)	6,951 (50.59%)	12,962 (69.09%)
Test of Significance			p=0.00	p=0.00		p=0.00	p=0.00
Vigorous activities							
Inactive	48,700 (68%)	34,641 (65.16%)	12,917 (55.41%)	21,696 (72.65%)	23,384 (71.97%)	8,667 (63.09%)	14,654 (78.13%)
Active	22,912 (32%)	18,519 (34.84%)	10,395 (44.59%)	8,166 (27.35%)	9,109 (28.03%)	5,070 (36.91%)	4,101 (21.87%)
Test of Significance			p=0.00	p=0.00		p=0.00	p=0.00
Smoking status							
Never	60,356 (84.28%)	44,254 (83.24%)	15,313 (65.7%)	28,885 (96.71%)	27,857 (85.74%)	9,535 (69.43%)	18,207 (97.07%)
Former	2,526 (3.53%)	1,741 (3.28%)	1,548 (6.64%)	206 (0.97%)	1,265 (3.89%)	1,141 (8.31%)	154 (0.83%)
Current	8,727 (12.19%)	7,166 (13.48%)	6,445 (27.66%)	776 (2.60%)	3,369 (10.37%)	3,057 (22.26%)	394 (2.10%)
Test of Significance			p=0.00	p=0.00		p=0.00	p=0.00
Chewing tobacco							
Never	55,783 (77.9%)	40,782 (76.71%)	15,609 (66.97%)	25,146 (84.19%)	25,653 (78.95%)	9,727 (70.82%)	15,869 (84.61%)
Former	1,665 (2.33%)	1,124 (2.12%)	731 (3.14%)	391 (1.33%)	878 (2.7%)	589 (4.29%)	300 (1.60%)
Current	14,161 (19.78%)	11,255 (21.17%)	6,968 (29.9%)	4,323 (14.48%)	5,959 (18.34%)	3,418 (24.89%)	2,587 (13.79%)
Test of Significance			p=0.00	p=0.00		p=0.00	p=0.00
Drinking status							
No	61,646 (86.06%)	45,258 (85.12%)	16,177 (69.39%)	29,032 (97.20%)	27,912 (85.87%)	9,463 (68.87%)	18,329 (97.68%)
Yes	9,984 (13.94%)	7,910 (14.88%)	7,135 (30.61%)	837 (2.80%)	4,592 (14.13%)	4,227 (31.13%)	434 (2.32%)
Test of Significance			p=0.00	p=0.00		p=0.00	p=0.00
Household factors							
MPCE quintile							
Poorest	14,955 (20.7%)	12,018 (22.47%)	5,166 (22.01%)	6,852 (22.82%)	6,005 (18.40%)	2,484 (18.0%)	3,518 (18.68%)
Poorer	15,328 (21.22%)	11,805 (22.07%)	5,111 (21.77%)	6,695 (22.29%)	6,513 (19.96%)	2,753 (19.95%)	3,760 (19.97%)
Middle	14,789 (20.47%)	10,944 (20.46%)	4,815 (20.51%)	6,132 (20.42%)	6,638 (20.34%)	2,740 (19.86%)	3,893 (20.68%)
Richer	14,151 (19.59%)	10,081 (18.85%)	4,462 (19.01%)	5,622 (18.72%)	6,644 (20.36%)	2,799 (20.28%)	3,844 (20.41%)
Richest	13,024 (18.03%)	8,647 (16.16%)	3,922 (16.71%)	4,729 (15.75%)	6,833 (20.94%)	3,024 (21.91%)	3,815 (20.26%)
Test of Signifficance			p=0.00	p=0.00		p=0.00	p=0.00
Religion							
Hindu	59,185 (81.92%)	44,613 (83.40%)	19,622 (83.57%)	25,004 (83.27%)	26,068 (79.88%)	11,075 (80.24%)	14,995 (79.63%)
Muslim	8,428 (11.67%)	5,588 (10.45%)	2,502 (10.66%)	3,089 (10.29%)	4,264 (13.07%)	1,701 (12.33%)	2,557 (13.58%)
Christian	2,142 (2.97%)	1,585 (2.96%)	574 (2.45%)	1,009 (3.36%)	966 (2.96%)	399 (2.89%)	566 (3.01%)
Others^$^	2,488 (3.44%)	1,705 (3.19%)	779 (3.32%)	926 (3.09%)	1,334 (4.09%)	626 (4.54%)	711 (3.78%)
Test of Significance			p=0.00	p=0.00		p=0.00	p=0.00
Caste							
Scheduled caste	13,688 (19.66%)	10,587 (20.48%)	4,647 (20.47%)	5,943 (20.48%)	5,708 (18.22%)	2,332 (17.56%)	3,371 (18.69%)
Scheduled tribe	6,101 (8.76%)	5,204 (10.07%)	2,201 (9.7%)	3,002 (10.35%)	2,287 (7.30%)	1,023 (7.70%)	1,266 (7.02%)
OBC^#^	32,526 (46.72%)	23,906 (46.23%)	10,485 (46.19%)	13,427 (46.26%)	14,877 (47.50%)	6,383 (48.05%)	8,479 (47.11%)
Others	17,309 (24.86%)	12,008 (23.22%)	5,364 (23.63%)	6,649 (22.91%)	8,450 (26.98%)	3,545 (26.69%)	4,903 (27.18%)
Test of Significance			p=0.00	p=0.00		p=0.00	p=0.00
Place of residence							
Rural	49,274 (68.2%)	38,809 (72.54%)	17,367 (73.97%)	21,458 (71.45%)	20,115 (61.64%)	8,612 (62.40%)	11,508 (61.11%)
Urban	22,975 (31.8%)	14,688 (27.46%)	6,111 (26.03%)	8,574 (28.55%)	12,519 (38.36%)	5,190 (37.60%)	7,324 (38.89%)
Test of Significance			p=0.00	p=0.00		p=0.00	p=0.00
Region							
North	8,674 (12.01%)	5,894 (11.02%)	2,690 (11.46%)	3,207 (10.68%)	4,371 (13.39%)	1,877 (13.60%)	2,495 (13.25%)
Central	14,521 (20.1%)	11,733 (22.01%)	5,676 (24.18%)	6,108 (20.34%)	5,448 (16.70%)	2,353 (17.05%)	3,097 (16.45%)
East	16,902 (23.4%)	12,968 (24.24%)	5,719 (24.36%)	7,253 (24.15%)	7,029 (21.54%)	2,996 (21.71%)	4,033 (21.42%)
Northeast	2,631 (3.64%)	1,900 (3.55%)	807 (3.44%)	1,093 (3.64%)	1,223 (3.75%)	532 (3.85%)	692 (3.68%)
West	11,906 (16.48%)	8,717 (16.30%)	3,695 (15.74%)	5,022 (16.72%)	5,763 (17.66%)	2,438 (17.67%)	3,324 (17.65%)
South	17,612 (24.38%)	12,242 (22.88%)	4,890 (20.83%)	7,347 (24.46%)	8,798 (26.96%)	3,604 (26.12%)	5,187 (27.55%)
Test of Significance			p=0.00	p=0.00		p=0.00	p=0.00
All (%)	100	72.83	43.45	56.55	45.16	41.02	58.97

Weighted estimation. ^#^Other Backward Classes; ^$^includes Sikh, Buddhist/neo-Buddhist, Jain, Parsi/Zoroastrian and others; ^a^ divorced, separated, and deserted; ^b^Activities of daily living includes dressing, walking across a room, bathing, eating difficulties, getting in or out of bed and toilet use (any one or more); ^c^ Instrumental Activities of Daily Living (IADL) includes preparing a hot meal, shopping for groceries, making telephone calls, taking medications, doing work around the house or garden, managing money and getting around or finding address in unfamiliar place (any one or more)

**Table 2:** Adjusted logistic regression showing individual characteristics associated with prevalence, and awareness of hypertension among males and females at 95%CI

	Prevalence		Awareness	
	Males	Females	Males	Females
Age Category(ref: 45–54 years)				
(55–64) years	1.43 (1.31–1.55)	1.40 (1.31–1.50)	1.31 (1.15–1.49)	1.12 (1.00–1.26)
(65–74) years	1.88 (1.71–2.07)	1.82 (1.68–1.97)	1.54 (1.33–1.79)	1.33 (1.17–1.52)
75 + years	2.06 (1.82–2.34)	1.86 (1.67–2.08)	1.74 (1.43–2.11)	1.36 (1.15–1.62)
Education Level (ref: no education)				
Primary	1.19 (1.09–1.30)	1.25 (1.16–1.34)	1.23 (1.07–1.40)	1.23 (1.09–1.37)
Secondary	1.27 (1.16–1.40)	1.10 (1.01–1.21)	1.35 (1.17–1.56)	1.11 (0.96–1.29)
Higher	1.46 (1.30–1.63)	0.99 (0.87–1.12)	1.51 (1.27–1.80)	0.99 (0.79–1.24)
Drinking alcohol status(ref: no)				
Yes	1.06 (0.98–1.14)	0.88 (0.75–1.03)	1.05 (0.94–1.18)	0.84 (0.66–1.03)
Chewing tobacco(ref: never)				
Former	1.02 (0.86–1.21)	1.14 (0.92–1.41)	0.97 (0.75–1.26)	1.24 (0.89–1.72)
Current	0.89 (0.82–0.97)	1.05 (0.97–1.14)	0.93 (0.82–1.06)	1.02 (0.90–1.16)
Smoking status(ref: never)				
Former	1.06 (0.94–1.18)	1.12 (0.88–1.43)	1.08 (0.90–1.28)	1.52 (1.01–2.29)
Current	0.90 (0.83–0.98)	0.90 (0.77–1.05)	0.86 (0.76–0.98)	0.80 (0.60–1.06)
Physical activity: vigorous(ref: inactive)				
Active	0.89 (0.83–0.96)	0.96 (0.90–1.03)	0.85 (0.76–0.95)	0.87 (0.77–0.98)
Physical activity: moderate(ref: inactive)				
Active	0.94 (0.88–1.01)	0.88 (0.82–0.93)	0.98 (0.88–1.09)	0.92 (0.83–1.01)
BMI categories(ref: normal)				
Underweight	0.62 (0.56–0.68)	0.64 (0.59–0.70)	0.66 (0.56–0.78)	0.67 (0.58–0.77)
Overweight/obese	1.68 (1.56–1.81)	1.81 (1.70–1.92)	1.54 (1.38–1.72)	1.63 (1.48–1.81)
Difficulty in IADL^c^ (ref: no)				
Yes	1.14 (1.05–1.23)	1.12 (1.05–1.19)	1.16 (1.02–1.32)	1.11 (1.00–1.22)
Difficulty in ADL^b^ (ref: no)				
Yes	1.28 (1.16–1.42)	1.15 (1.07–1.25)	1.30 (1.11–1.53)	1.21 (1.07–1.37)
Arthritis (ref: no)				
Yes	1.32 (1.17–1.49)	1.42 (1.31–1.55)	1.36 (1.12–1.64)	1.51 (1.31–1.79)
Stroke (ref: No)				
Yes	3.60 (2.96–4.38)	2.97 (2.32–3.79)	3.57 (2.58–4.95)	2.95 (2.01–4.34)
Diabetes (ref: No)				
Yes	3.65 (3.37–3.97)	3.46 (3.21–3.74)	3.88 (3.41–4.40)	3.63 (3.20–4.13)
Working status(ref: never worked)				
Currently working	0.80 (0.68–0.94)	0.79 (0.73–0.85)	0.89 (0.69–1.15)	0.76 (0.67–0.87)
Not currently working	1.13 (0.96–1.33)	1.04 (0.97–1.12)	1.21 (0.94–1.56)	1.05 (0.94–1.17)
Marital status (ref: currently married)				
Widowed	0.98 (0.88–1.09)	1.29 (1.22–1.38)	1.10 (0.94–1.29)	1.19 (1.07–1.31)
D/S/D/others^a^	0.84 (0.69–1.02)	1.07 (0.92–1.24)	0.68 (0.50–0.92)	1.06 (0.83–1.35)
Religion (ref: Hindu)				
Muslim	1.10 (0.99–1.22)	1.35 (1.24–1.47)	1.22 (1.03–1.43)	1.31 (1.14–1.51)
Christian	0.97 (0.84–1.11)	1.02 (0.91–1.14)	0.91 (0.74–1.11)	1.03 (0.86–1.23)
Others^$^	1.11 (0.96–1.28)	1.16 (1.02–1.31)	1.03 (0.83–1.22)	1.07 (0.88–1.32)
Caste (ref: scheduled caste)				
Scheduled tribe	0.80 (0.70–0.91)	0.75 (0.67–0.83)	0.77 (0.64–0.94)	0.73 (0.61–0.86)
OBC^#^	0.98 (0.89–1.08)	1.00 (0.92–1.08)	0.94 (0.81–1.10)	1.05 (0.92–1.20)
Others	1.07 (0.97–1.19)	1.03 (0.95–1.13)	1.09 (0.93–1.27)	1.00 (0.87–1.15)
Place of residence (ref: rural)				
Urban	1.21 (1.13–1.30)	1.21 (1.14–1.29)	1.14 (1.03–1.28)	1.20 (1.09–1.33)
Region (ref: north)				
Central	0.70 (0.62–0.79)	0.67 (0.60–0.74)	0.68 (0.56–0.84)	0.48 (0.40–0.57)
East	0.91 (0.81–1.02)	0.79 (0.72–0.87)	1.05 (0.88–1.25)	0.71 (0.61–0.83)
Northeast	1.15 (1.00–1.32)	0.92 (0.82–1.04)	1.35 (1.10–1.65)	0.98 (0.81–1.19)
West	0.86 (0.77–0.97)	0.74 (0.67–0.82)	0.80 (0.66–0.96)	0.62 (0.52–0.73)
South	1.00 (0.90–1.11)	0.80 (0.73–0.87)	0.99 (0.85–1.16)	0.69 (0.59–0.79)
MPCE quintile (ref: poorest)				
Poorer	1.09 (0.98–1.21)	1.20 (1.09–1.31)	1.17 (1.00–1.38)	1.28 (1.11–1.47)
Middle	1.16 (1.04–1.28)	1.32 (1.21–1.44)	1.20 (1.02–1.41)	1.31 (1.14–1.51)
Richer	1.28 (1.15–1.42)	1.40 (1.29–1.53)	1.25 (1.07–1.47)	1.32 (1.15–1.53)
Richest	1.36 (1.22–1.51)	1.44 (1.32–1.58)	1.33 (1.12–1.57)	1.43 (1.23–1.66)

Weighted estimation. ^#^Other Backward Classes; ^$^includes Sikh, Buddhist/neo-Buddhist, Jain, Parsi/Zoroastrian and others; ^a^ divorced, separated, and deserted; ^b^Activities of daily living includes dressing, walking across a room, bathing, eating difficulties, getting in or out of bed and toilet use (any one or more); ^c^ Instrumental Activities of Daily Living (IADL) includes preparing a hot meal, shopping for groceries, making telephone calls, taking medications, doing work around the house or garden, managing money and getting around or finding address in unfamiliar place (any one or more)

**Table 3:** Adjusted logistic regression showing individual characteristics associated with treatment and control of hypertension among males and females at 95%CI

	Treatment		Control	
	Males	Females	Males	Females
Age category (ref: 45–54 years)				
(55–64) years	2.44 (1.31–4.55)	1.30 (0.82–2.05)	0.96 (0.83–1.01)	0.86 (0.77–0.96)
(65–74) years	2.14 (1.13–4.07)	2.06 (1.16–3.65)	0.96 (0.82–1.12)	0.73 (0.65–0.83)
75 + years	1.94 (0.82–4.57)	1.37 (0.66–2.81)	0.82 (0.67–1.01)	0.59 (0.49–0.69)
Education Level (ref: No education)				
Primary	1.48 (0.83–2.63)	1.51 (0.89–2.56)	0.90 (0.78–1.05)	1.03 (0.92–1.14)
Secondary	1.85 (0.97–3.53)	2.26 (0.97–5.24)	0.83 (0.71–0.97)	0.98 (0.86–1.13)
Higher	2.68 (1.04–6.93)	2.78 (0.62–12.40)	0.88 (0.73–1.05)	1.12 (0.92–1.37)
Drinking alcohol status (ref: no)				
Yes	0.85 (0.50–1.45)	1.98 (0.41–9.49)	0.70 (0.62–0.79)	0.86 (0.66–1.13)
Chewing tobacco (ref: never)				
Former	1.33 (0.36–4.88)	0.63 (0.17–2.31)	1.18 (0.90–1.54)	0.82 (0.59–1.13)
Current	0.81 (0.45–1.44)	2.18 (1.05–4.51)	0.95 (0.82–1.10)	0.91 (0.80–1.04)
Smoking status (ref: never)				
Former	1.25 (0.51–3.04)	0.14 (0.06–0.33)	1.12 (0.93–1.34)	1.11 (0.76–1.61)
Current	1.01 (0.54–1.88)	0.28 (0.14–0.55)	1.20 (1.04–1.39)	1.54 (1.16–2.03)
Physical activity: vigorous (ref: inactive)				
Active	0.86 (0.51- 1.47)	0.82 (0.51–1.32)	1.11 (0.98–1.25)	1.10 (0.98–1.24)
Physical activity: moderate (ref: inactive)				
Active	0.82 (0.49–1.36)	0.61 (0.39–0.96)	0.90 (0.80–1.01)	0.85 (0.77–0.94)
BMI categories (ref: normal)				
Underweight	0.36 (0.20–0.64)	0.94 (0.57–1.54)	1.61 (1.34–1.94)	1.31 (1.13–1.53)
Overweight/obese	1.49 (0.82–2.72)	3.04 (1.88–4.93)	0.74 (0.66–0.83)	0.77 (0.70–0.84)
Difficulty in IADL^c^ (ref: no)				
Yes	0.71 (0.41–1.20)	0.84 (0.56–1.26)	0.96 (0.84–1.09)	1.01 (0.92–1.11)
Difficulty in ADL^b^ (ref: no)				
Yes	2.20 (1.01–4.79)	1.15 (0.67–1.99)	0.99 (0.84–1.15)	0.88 (0.79–0.99)
Arthritis (ref: no)				
Yes	0.68 (0.29–1.60)	1.33 (0.64–2.72)	1.02 (0.85–1.23)	0.97 (0.86–1.01)
Stroke (ref: no)				
Yes	3.38 (0.72–15.84)	1.96 (0.42–9.14)	1.28 (1.01–1.62)	0.80 (0.61–1.05)
Diabetes (ref: no)				
Yes	3.96 (1.66–9.44)	3.09 (1.53–6.22)	0.95 (0.85–1.07)	0.86 (0.78–0.95)
Working status (ref:never worked)				
Currently working	0.41 (0.11–1.52)	0.73 (0.43–1.22)	0.91 (0.70–1.20)	1.14 (1.01–1.30)
Not currently working	0.62 (0.16–2.34)	0.82 (0.49–1.36)	0.89 (0.68–1.16)	0.96 (0.87–1.07)
Marital status (ref: currently married)				
Widowed	1.41 (0.65–3.08)	1.65 (1.05–2.59)	0.68 (0.57–0.81)	0.79 (0.72–0.87)
D/S/D/others^a^	0.37 (0.10–1.32)	0.44 (0.18–1.08)	1.18 (0.85–1.65)	0.82 (0.65–1.05)
Religion (ref: Hindu)				
Muslim	0.90 (0.41–1.98)	1.01 (0.55–1.84)	0.75 (0.63–0.89)	0.84 (0.74–0.95)
Christian	0.13 (0.03–0.50)	0.14 (0.04–0.46)	1.03 (0.83–1.28)	0.94 (0.79–1.31)
Others^$^	0.19 (0.09–0.41)	0.19 (0.10–0.35)	0.76 (0.60–0.96)	0.90 (0.75–1.09)
Caste (ref: scheduled caste)				
Scheduled tribe	1.55 (0.54–4.41)	0.90 (0.37–2.13)	0.77 (0.62–0.96)	0.76 (0.64–0.91)
OBC^#^	1.28 (0.70–2.35)	0.90 (0.54–1.50)	1.11 (0.95–1.30)	1.11 (0.98–1.26)
Others	1.67 (0.88–3.17)	1.04 (0.60–1.80)	1.08 (0.91–1.28)	1.03 (0.90–1.18)
Place of residence (ref: rural)				
Urban	4.00 (1.97–8.14)	2.08 (1.25–3.44)	1.05 (0.93–1.17)	1.07 (0.97–1.18)
Region (ref: north)				
Central	0.76 (0.34–1.69)	0.83 (0.44–1.56)	1.25 (1.01–1.54)	1.43 (1.20–1.69)
East	0.50 (0.25–1.01)	0.59 (0.36–0.99)	1.01 (0.84–1.21)	0.99 (0.85–1.14)
Northeast	4.98 (1.44–17.22)	5.90 (1.77–19.60)	0.73 (0.58–0.91)	0.83 (0.68–1.01)
West	1	6.90 (2.04–23.24)	1.17 (0.97–1.41)	1.26 (1.08–1.47)
South	53.15 (6.54–432.02)	1	0.93 (0.79–1.10)	0.95 (0.83–1.08)
MPCE quintile (ref: poorest)				
Poorer	1.87 (0.83–4.24)	2.10 (1.18–3.75)	0.90 (0.75–1.08)	1.03 (0.89–1.19)
Middle	1.64 (0.75–3.58)	2.49 (1.40–4.42)	0.93 (0.78–1.11)	1.05 (0.91–1.21)
Richer	1.18 (0.57–2.44)	2.49 (1.14–4.40)	1.05 (0.88–1.26)	1.17 (1.02–1.35)
Richest	1.53 (0.70–3.35)	2.52 (1.38–4.58)	1.17 (0.98–1.40)	1.21 (1.04–1.39)

^#^Other Backward Classes; ^$^includes Sikh, Buddhist/neo-Buddhist, Jain, Parsi/Zoroastrian and others; ^a^ divorced, separated, and deserted; ^b^Activities of daily living includes dressing, walking across a room, bathing, eating difficulties, getting in or out of bed and toilet use (any one or more); ^c^ Instrumental Activities of Daily Living (IADL) includes preparing a hot meal, shopping for groceries, making telephone calls, taking medications, doing work around the house or garden, managing money and getting around or finding address in unfamiliar place (any one or more)

The multivariable logistic regression model that was implemented can be explained as follows: –

$$\mathrm{Log} \left(\frac{\pi i}{1-\pi i}\right)=\upbeta_{0} +\upbeta_{1}\mathrm{X}_{1} +\upbeta_{2}\mathrm{X}_{2} + \cdots \mathrm{ \beta_{n}X_{n}}$$

where π(*x*) = *P*(*Y* = *1|X* = *x*) is a binary independent variable *Y* with two categories, p = probability of an event (Prevalence, Awareness, Treatment, Control of Hypertension), βi = regression coefficients associated with the reference group, Xi = explanatory variables. (Independent variables at Socio-demographic, Lifestyles, Health-status), The reference group, represented by β0, is constituted by those individuals presenting the reference level of each and every variable X1…i.

## Results

Our sample contains 72,250 people with a mean age of 57.9 years (SD 11.6), out of whom 42% were male and 58% female (Table 1). Overall, 45.1% of the study population was found to be hypertensive, of which 26.9% self-reported their hypertension and 30% were found to be hypertensive at the time of measurement (Table 1). About 41% of males and 59% of females were found to be hypertensive. Table 1 reveals that among all hypertensive females falls highest proportion belongs to 55–64 age group. However when it comes to males, the majority of hypertensive cases are observed in a wider age bracket, specifically between 45–64 age group. Males with high blood pressure were more likely to be 45–54 years old, have lower educational status, be currently employed, have a normal BMI, be inactive in vigorous activities, belong to the richest MPCE quintile, live in rural areas, and be from the southern regions. Whereas females with high blood pressure were more likely to be between the ages of 55 and 64 years, have a lower educational status, never work, possess a normal BMI, be inactive in vigorous activities, be from the middle MPCE quintile, and live in the southern region of India (Table 1). Men's self-reported hypertension was found to differ noticeably from measured hypertension by 8.7%, whereas in women it was only 1.2%.

Males and females not having hypertension were more likely to come from the poorest quintile and eastern region, whereas those with hypertension belong to the richest quintile and southern region. In the hypertensive population, proportion of males who had never worked were 3.0%, while females were 48.7% (Table 1). Additionally, we discovered that both hypertensive males and females had higher proportion of not smoking, using tobacco, or drinking alcohol. The BMI is one of the essential lifestyle characteristics in hypertensive males; 54.4% were in the normal category, 30.6% were found to be overweight or obese, and 14.5% were underweight. In hypertensive females, 43.7% were in the normal category, 42.0% were found to be overweight or obese, and 14.2% were underweight. 

**Fig. 1:**
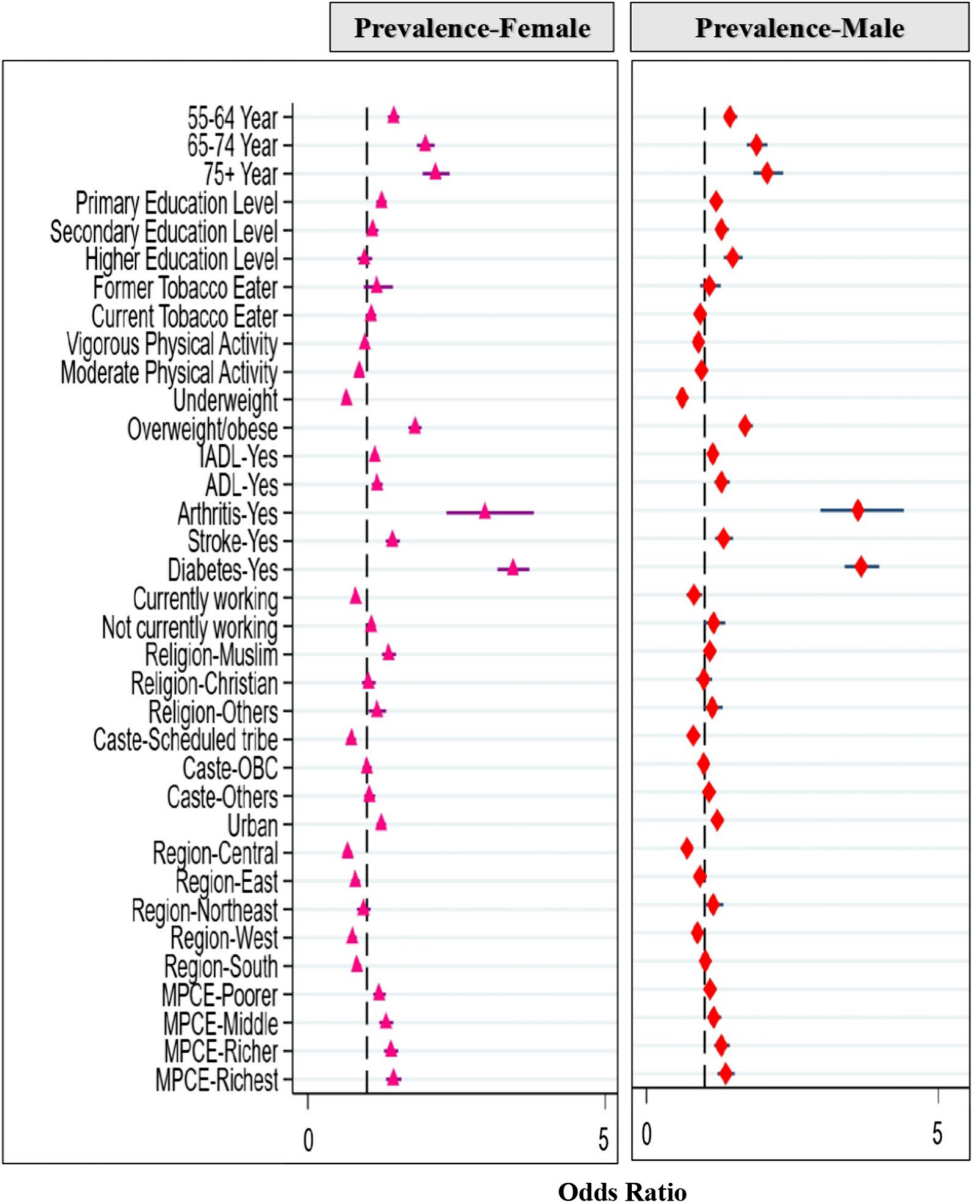
The determinants of the prevalence of hypertension among females (left) and males (right) in multivariable analysis in India

Various variables linked to hypertension prevalence, awareness, treatment, and control were identified using multivariable models. Table 2 shows that increasing age was significantly associated with an increased risk of hypertension in males and females. But the rate of increase in prevalence was higher in males than in females. Males with a higher educational level were more likely to have hypertension as compared to males with no education, whereas females with a higher educational status were not significantly associated with hypertension (Fig. 1). Drinking alcohol, being a former smoker, or chewing tobacco were not significantly associated with hypertension for both males and females. Being physically active and participating in vigorous activity was a protective factor in hypertension prevalence for males. Being physically active and engaging in moderate activity was a protective factor for females. Being underweight lowers the chance of having hypertension in both males and females (AOR = 0.62, 95% CI-(0.56–0.68) and AOR = 0.64, 95% CI-(0.59–0.70)) as compared to normal-weight adults, respectively, whereas being overweight increases the risk of having hypertension in both males and females (AOR = 1.68, 95% CI-(1.56–1.81) and AOR = 1.81, 95% CI-(1.70–1.92), respectively, as compared to normal-weight adults. Diabetes increased the odds of having hypertension in males (AOR = 3.65, 95% CI 3.37–3.97) and in females (AOR = 3.46, 95% CI 3.21–3.74) (Fig. 1). Both males and females were more likely to have hypertension if they were residents of urban areas and in the richest MPCE quintile. Muslim females were more likely to have hypertension in comparison with Hindu females (AOR = 1.35, 95% CI 1.24–1.47).

**Fig. 2:**
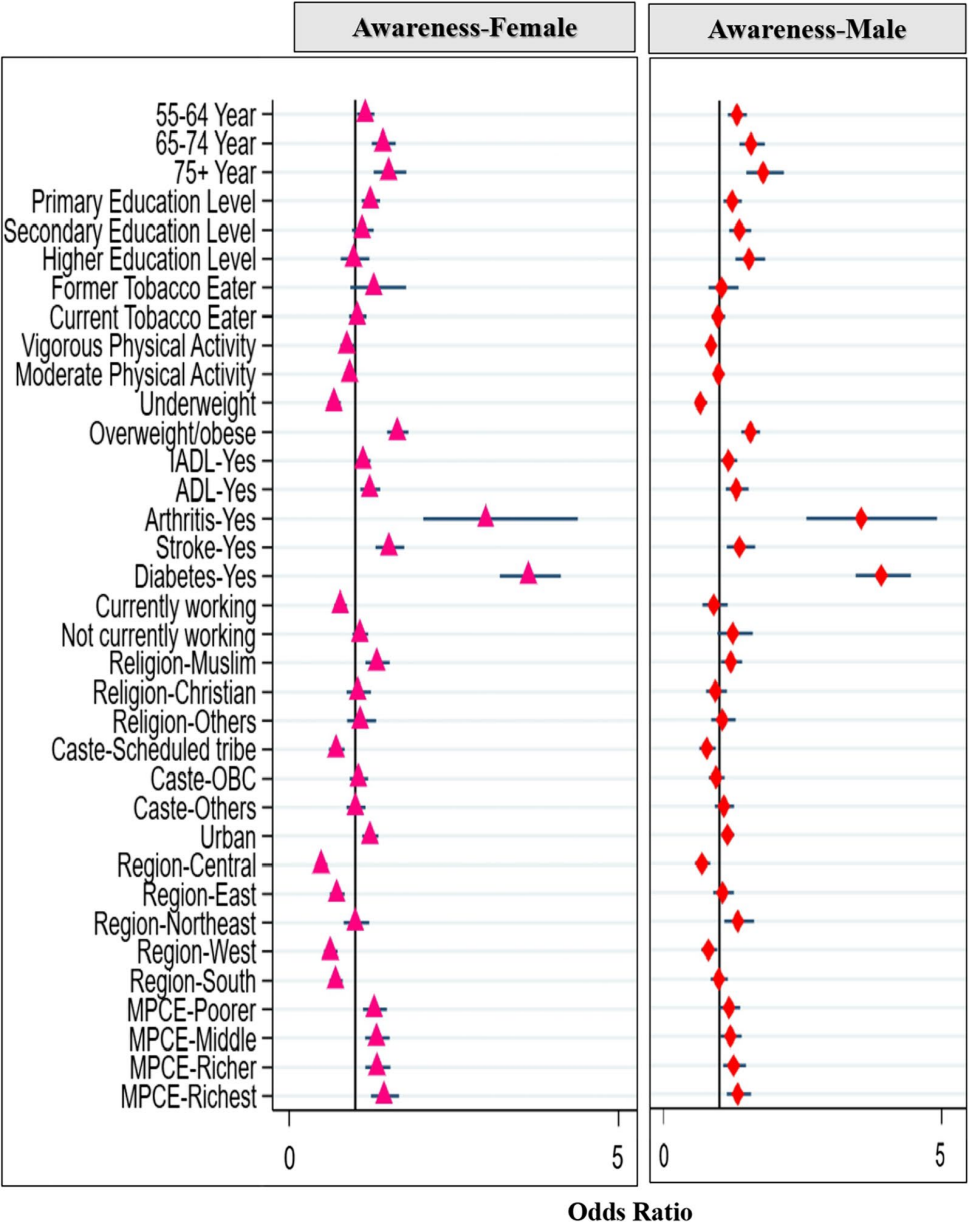
The determinants of awareness of hypertension among females (left) and males (right) in multivariable analysis in India

Males and females in the 75 + age groups were more likely to be aware of hypertension as compared to other age groups. Males with a higher educational degree were more likely to be aware of hypertension. Females who were former smokers were more likely to be aware of hypertension as compared to never-smokers (Fig. 2). BADL_s_, diabetes, arthritis, and stroke patients (males and females) are more likely to be aware of hypertension than those who are not. Males and females who were overweight or obese (AOR = 1.54, 95% CI 1.38–1.72) and AOR = 1.63, 95% CI 1.48–1.81) were more likely to be aware of hypertension than those who were of normal weight. Underweight males and females were less likely to be aware of hypertension (AOR = 0.66, 95% CI 0.56–0.78, and AOR = 0.67, 95% CI 0.58–0.77) as compared to those of normal weight (Fig. 2). Muslim males and females were more aware of hypertension as compared to Hindus (AOR = 1.22, 95% CI 1.03–1.43) and Hindus (AOR = 1.31, 95% CI 1.14–1.51), respectively. Males and females in the richest MPCE quintiles were more likely to be aware of hypertension than those in the poorest quintiles.

The likelihood of seeking treatment for hypertension declines with increasing age among males. However, there was a robust connection in females for the age range 65–74 years (AOR = 2.06, 95% CI: (1.16–3.65)). In our study, males with higher educational levels were more likely to be taking hypertension treatment as compared to males with no education. The study found that among females, those who currently chew tobacco were more likely to be receiving treatment for hypertension compared to those who had never chewed tobacco. However, females who were former or current smokers were less likely to be receiving treatment for hypertension compared to non-smoking females. Additionally, being overweight or obese and belonging to the highest quintile of the MPCE were found to increase the likelihood of receiving treatment for hypertension among females. Both males and females were more likely to seek treatment for hypertension if they had diabetes and resided in urban areas.

As females aged, they were less likely to have hypertension under control. Males who consumed alcohol had a lower likelihood of controlling their hypertension than non-drinkers. Surprisingly, we found that both males and females who were current smokers and underweight were more likely to keep their blood pressure under control than never-smokers and normal-weight adults, respectively. Females with diabetes were less likely to have their blood pressure under control as compared to non-diabetic females. Females in the richest quintile (OR = 1.21, 95% CI 1.04–1.39) were more likely to have their hypertension under control as compared to the poorest quintile females.

## Discussions

This study is one of the first to use nationally representative data from the Longitudinal Ageing Study in India (LASI) to analyse the sex disparities in hypertension prevalence, awareness, treatment, and control, and their associated factors with hypertension among adults in India. The findings revealed that for males, the factors most strongly associated with hypertension were being in the age group of 45–54 years, having no education, being currently employed, currently married, being overweight, being inactive in vigorous physical activities, never having smoked or chewed tobacco, being diabetic, having a history of stroke, being in the richest quintile, residing in rural areas, and living in the southern region of India. For females, the factors most strongly and significantly associated with hypertension were being in the age group of 55–64 years, having no education, never having worked, being currently married, being overweight, being widowed, being Muslim, being diabetic, having a history of stroke, being inactive in vigorous physical activities, and residing in India.

In this research, the prevalence of ALL hypertension was 45.1%, with males accounting for 41% and females accounting for 58.9%. However NFHS-4 India study found the total prevalence of measured hypertension in males aged 15–54 years was 16.3% and in females aged 15–49 years was 11.5% [21]. WHO findings of 2019 globally for 30–79-year-old adults: hypertension in males was 30.6% and in females was 29.5% [22], a research conducted in Varanasi, the prevalence of measured hypertension was found to be 40.6% in males and 26.0% in females [23]. According to these studies, the prevalence of measured hypertension was found to be significantly higher in males compared to females. However, it is difficult to pinpoint a specific explanation for this observation as various factors may contribute to the findings. One possible explanation for the higher prevalence of self-reported hypertension among males could be that in the past, men were typically the primary breadwinners and had more access to healthcare and resources, which may have resulted in increased stress and workload.

Our study found that individuals aged 75 and above, both males and females, have significantly lower treatment and control of hypertension compared to those aged 45 to 54 years. This could be due to several reasons, such as older people being more dependent on others for their care as well as the fact that hypertension often lacks noticeable symptoms, making it more difficult for older individuals to detect and manage the disease. This is why hypertension is often referred to as the "silent killer". A study done by Zhang & Moran [24], demonstrates that youngsters (18–39 years old) are more likely than their older counterparts (40 + years old) to acquire BP control with treatment. Males were more aware of hypertension than females in the 45–54 age range, while female treatment and control of hypertension were considerably more significant than males for the same age group. Previous studies have shown that "infrequent healthcare visits are an important risk factor for low awareness and management" [25]. Our study revealed that Muslim females were more likely to have hypertension and be more aware of it, but less likely to effectively manage it compared to Hindu females. In contrast, there was no significant relationship between religion and hypertension among males. However, Muslim males were more aware of the disease but less likely to control it compared to Hindu males. This higher prevalence of hypertension among Muslim females than Hindu females may be partly attributed to cultural beliefs and dietary patterns that influence the lifestyle of Muslim females [21]. Previous research has found an association between the consumption of non-vegetarian foods and hypertension, whereas a vegetarian diet has been shown to have a protective effect against hypertension [23]. In our study, females in the richest MPCE households had a higher prevalence, awareness, treatment, and control of hypertension than the poorest sections.

Our study found that males with higher levels of education were more likely to be aware of hypertension compared to those with no education. However, our findings also revealed that men with higher education had a greater risk of hypertension than men with no education. This could be because men with higher education tend to work primarily as professionals may have a more sedentary lifestyle, lack regular physical activity, and consume foods high in fat and sodium. On the other hand, another study found that women with higher levels of education had a lower risk of hypertension than women with no education [25]. Previous research [5, 24] has found that individuals with higher levels of education tend to have higher blood pressure compared to those with lower levels of education. However, other studies have also shown that highly educated women have a better understanding of healthcare, which can result in a decreased risk of hypertension, which is contradictory to our findings. This highlights the complexity of how education and hypertension are related and that other factors may also play a role [29]. Males in the highest income bracket, or richest quintile, had a higher prevalence and greater awareness of hypertension compared to those in the lowest income bracket, or poorest quintile. These findings are consistent with previous research that suggests that individuals with higher income levels have greater access to healthcare and better education and may experience more stress due to their occupation compared to lower-income individuals. This access to healthcare and education may also help them be more aware of hypertension and its management [17, 24, 26].

Our study found that widowed females were more likely to have hypertension, be aware of it, and seek treatment compared to married females. However, they were less likely to effectively manage their hypertension. This could be due to the challenges of living alone at the age of 45 or older, particularly for women who may have relied on their partner for support. This is consistent with previous research, which has found that married individuals have a lower prevalence of hypertension compared to single, divorced, or widowed individuals [28]. Surprisingly, our study did not find an association between smoking and drinking alcohol with the risk of hypertension in both males and females. One possible explanation is that as smoking and drinking alcohol are still stigmatised in Indian society, this could have led to underreporting of these behaviours. This is consistent with a previous study conducted in Korea which also found that smoking did not affect hypertension treatment in either males or females [31]. Our study found that among females, former smokers and current tobacco chewers were more likely to be aware of hypertension and to seek treatment for it. Additionally, we found that among males, alcohol consumption was associated with a lower likelihood of effectively managing hypertension. These findings contrast with previous studies that have consistently shown a strong association between alcohol intake and a high prevalence of hypertension in both males and females [19, 24, 25].

In this study, we found that being underweight was associated with a lower prevalence of hypertension in both males and females, while being overweight or obese increased the risk of hypertension, seeking treatment, and awareness of hypertension in both males and females. Our findings also revealed that underweight individuals were more likely to have better blood pressure control compared to those of normal weight. These results are consistent with previous research that has examined the relationship between body mass index (BMI) and hypertension [28, 29]. The results of this study found that for males, being physically fit as measured by participation in vigorous activity was associated with a lower risk of hypertension. However, this association was not significant among females. Although we discovered that, among females, engaging in moderate physical activity was linked to a decreased likelihood of developing hypertension, These findings align with previous research that has reported a positive association between physical activity and hypertension control in both sexes [30, 31]. Our study found that diabetes was strongly associated with hypertension prevalence, awareness, and treatment in both males and females. However, diabetic females were less likely to effectively manage their hypertension compared to non-diabetic females. Both diabetic males and females were more likely to have hypertension, be aware of it, and seek treatment [36]. Our study is in line with previous research that has found that diabetic men and women have a higher prevalence of hypertension than non-diabetic individuals. However, findings also suggest that diabetic women have an even greater risk of hypertension compared to non-diabetic women [17, 33, 34]. A study suggests that diabetes, which is known to be associated with hypertension, may be caused by unhealthy dietary habits, a sedentary lifestyle, and a lack of physical activity. These are established risk factors for hypertension and are likely to contribute to the development of both diabetes and hypertension [39].

In our study, both currently employed males and females were found to have a lower prevalence of hypertension compared to their non-employed counterparts. Additionally, the results indicated that currently employed females were more likely to effectively manage their hypertension than males. However, these findings contradict previous studies on older adults, which only found a relationship between hypertension treatment and control in relation to the employment status of males [36, 37]. WHO has recommended the implementation of workplace-based wellness programmes as a strategy to address hypertension [42]. Interestingly, our analysis found no significant relationship between unemployment and hypertension, which contrasts with the findings of previous studies conducted in South Korea, which have identified unemployment as a risk factor for poor hypertension management among women [43]. Previous research has shown that unemployment can limit women's access to regular medical check-ups and facilities, increasing their likelihood of developing hypertension. Our study found that hypertension was more prevalent among urban males and females compared to rural inhabitants and that they were more likely to be aware of their condition and seek treatment. The higher incidence of hypertension in urban populations may be attributed to factors such as busy lifestyles, a lack of physical activity, and stressful environments commonly found in urban areas [21]. In India, awareness of healthcare, health-seeking behaviours, and access to quality health services, particularly among rural women, is still quite limited [44].

### Study’s strength and limitation

Our research advanced upon previous studies by using the newly published LASI data, which enabled us to estimate hypertension prevalence at multiple geographic levels. Multivariate analysis was employed to identify the primary determinants of hypertension in India. However, we also identified limitations in our research. For example, previous studies had inconsistent definitions of hypertension, making comparisons difficult. Additionally, qualitative analysis is needed to fully understand the sex disparity in hypertension prevalence, awareness, treatment, and control. There is no data available on the frequency of healthcare visits. The cross-sectional design of the study raises the possibility of causal inference, but we were unable to gather information on patients' medication adherence, which limits our ability to study potential causes of inadequate treatment. Additionally, the data was self-reported, which is susceptible to social desirability, recall bias, and underreporting. Another limitation is that we did not inquire about the list of participants taking antihypertensive medicines, which could have assisted us in estimating the prevalence of resistant hypertension in this group. Furthermore, the LASI survey took blood pressure at the participants' homes during a single visit, although it was collected three times in a single visit, which can lead to higher average readings, an overestimation of the incidence of hypertension, and an incorrect assessment of patient awareness of the condition and the effectiveness of treatment [45].

## Conclusions

Our study found that among Indian adults, 45.1% had hypertension. The self-reported hypertension of men was found to differ noticeably from measured hypertension by 8.7%, whereas in women it was only 1.2%. The results of our study showed that physical activity has a protective effect against hypertension in both males and females. Therefore, it is possible to establish health promotion campaigns aimed at encouraging individuals to participate in physical activity. Education policies should focus on promoting healthy lifestyles among educated males. Muslim females are more likely to have hypertension, and public policies could address this through targeted interventions. Diabetes increases the likelihood of hypertension in both males and females, and public policies could focus on improving chronic disease management. Being underweight lowers the risk of hypertension, while being overweight increases it, so public policies could focus on improving nutrition. Elderly individuals are more likely to be aware of hypertension, so public policies could improve healthcare services for this population, including hypertension screening and management. Based on our findings, we can recommend sex based and population-based methods that need to be addressed for policy implications.

## Data Availability

The study uses secondary data which is available on request through https://www.iipsindia.ac.in/content/LASI-data.
